# Evaluating disparity of subjective cognitive decline between male veterans and non-veterans in the United States using propensity score matching estimation: A behavioral risk factor surveillance system survey cross-sectional study

**DOI:** 10.1371/journal.pone.0310102

**Published:** 2024-09-13

**Authors:** Chinenye Ifebirinachi, Man Sik Park, Seong-Tae Kim

**Affiliations:** 1 Department of Mathematics and Statistics, North Carolina Agricultural & Technical State University, Greensboro, North Carolina, United States of America; 2 Department of Statistics, Sungshin Women’s University, Seoul, Republic of Korea; Ajou University School of Medicine and Graduate School of Medicine, REPUBLIC OF KOREA

## Abstract

Subjective cognitive decline (SCD) is one of the most important early onset symptoms of Alzheimer’s Disease. Previous studies consistently reported that SCD is associated with quality of life, socio-economic factors, and related health comorbidities. However, the impact of veteran status on SCD has been little investigated. This study conducted a cross-sectional study to address disproportionate effects in subjective cognitive decline between veterans and non-veterans in the United States. Propensity score matching (PSM) was applied in this observational study to achieve covariate balancing and reduce selection bias, providing a more accurate estimate of the isolated effect of veteran status on SCD. Our study utilized 32,431 forty-five years or older non-institutionalized White, Black or African-American, and Hispanic or Latin-American male population from the 2019 Behavioral Risk Factor Surveillance System data. We first identified 10,685 paired PSM samples for the binary veteran status using the preselected covariates. Next, we performed a logistic regression for modeling the relationship between the veteran status and the SCD status using the PSM samples along with the covariates selected by a BIC-based stepwise selection. Our analyses revealed a statistically significant causal association between veteran status and SCD after PSM (odds ratio (OR): 1.16 and 95% confidence interval (CI): 1.06–1.27). We obtained a similar result before PSM with an OR of 1.20 and 95% CI of 1.10–1.31. When we focused on a minority group (Black or African-American males), we found a significantly increased veterans’ risk of SCD, especially after propensity score matching (OR: 1.69, 95% CI: 1.16–2.45). We also found several factors such as employment status, difficulty dressing/walking/running errands, general health status, physical health status, unaffordability of medical costs, mental health status, and comorbid conditions including stroke, blindness, high cholesterol, and arthritis as statistically significantly associated with SCD (*P-value* < 0.05). Similar to post-traumatic stress disorder and traumatic brain injury, our study demonstrated a causal association between SCD and military-related activities in the United States, which has a disproportionate impact on the minority population. This study sets the groundwork to further research in this domain to diagnose neurological diseases early among veterans.

## Introduction

Subjective Cognitive Decline (SCD) is a self-reported, persistent decline in cognition, which is not verified by neuropsychological testing and is potentially an early indicator of Alzheimer’s disease (AD) and other dementias [1]. By 2050, there will be an estimated 66 million cases of dementia worldwide, in which AD will comprise up to 80% of diagnosed dementia cases [2,3]. Recent research has shown that AD is significantly associated with multiple risk factors such as hypertension, obesity, diabetes, depression, physical inactivity, lack of education, smoking, high cholesterol, and other risk factors [4–8]. The risk factors for SCD, an early sign of AD and dementia, overlap with those for AD. Studies have linked SCD with depression, anxiety, medication use, substance use, body mass index (BMI), and smoking [1,9]. SCD’s association with an increased potential risk of dementia underscores its population-level estimates crucial for health policy and public health decision-making [10]. Many health-related and lifestyle risk factors, potentially modifiable during and after active military service, may originate from military-related activities [5].

Mental health research in active military personnel and veterans has predominantly focused on post-traumatic stress disorder (PTSD) and traumatic brain injury (TBI). For instance, in 2010, the Centers for Disease Control and Prevention estimated the medical costs for domestic TBI at approximately $76.3 billion [11]. Notably, 11 to 22 percent of veterans suffer from PTSD, compared to 6 percent of non-veterans in the United States. Recently, the United States Department of Defense (DoD) and the Department of Veterans Affairs (VA) have funded several research projects investigating the prevalence of PTSD and TBI [11]. Studies have found that PTSD and TBI increase the risk of dementia among veterans [12]. Schmeidler et al. [9] investigated the relationship between BMI and cognition in cognitively healthy aged veterans. Research on SCD in veterans remains limited despite its connections to dementia and AD.

This study aims to estimate the effect of veteran status on subjective cognitive decline using the 45 years or older White, Black or African-American, Hispanic or Latin-American male population obtained from the 2019 Behavioral Risk Factor Surveillance System (BRFSS) public data. Hereafter, all population denotes the White, Black or African-American, Hispanic or Latin-American racial groups. Although Olivari et al. [10] estimated the prevalence of SCD across the 50 states in the United States using the 2018 state-based BRFSS data, there is a lack of studies examining SCD in the veteran population. Furthermore, we perform a subset analysis of the relationship between the two factors using the Black or African-American population to investigate if there is any disproportionality in this minority population. This subset analysis overlaps and expands prior research that addressed diversity and disparity in minority mental health [13].

Multiple studies have utilized various annual editions of the BRFSS public data, which assess the prevalence of specific behavioral and health conditions, primarily relying on basic statistical techniques or association analyses. For example, a study using 1987–2000 BRFSS data, Simpson et al. [14] focused on investigating walking trends among adults in 31 U.S. states, reporting descriptive statistics such as frequencies, percentages, and 95% confidence intervals. Similarly, Kim and Beckles [15] used t-tests and chi-square tests to compare cardiovascular disease risk status and gender differences, aiming to examine suboptimal cardiovascular disease risk-reduction practices in high-risk populations, especially regarding gender disparities. Chou et al. [16] complemented BRFSS data with state-level metrics on fast-food and full-service restaurants, food consumption at home, cigarette usage, alcohol consumption, and clean indoor air regulations to investigate factors contributing to the rise in obesity in the U.S. since the late 1970s. Strine et al. [17] studied the relationships between depression and anxiety and the selected behavioral risk factors, obesity, and chronic diseases at the state level. Another study, assessing the adoption of computed tomography screening, used counts and 95% confidence intervals to analyze data from the 2017 BRFSS across ten states, prompted by findings from the National Lung Screening Trial [18].

The BRFSS survey data differs from randomized controlled trial (RCT) experiments. In RCTs, subjects are categorized into treated and control groups based on treatment effect, randomly balancing measured and unmeasured baseline covariates across groups [19]. This kind of balance is absent in observational studies, but estimating causal effects still necessitates comparing potential outcomes in exposed, equivalent to treated in RCTs, and unexposed groups [20]. Researchers have adopted various statistical approaches, such as marginal structural models, to address this limitation in observational studies. Our study implements propensity score estimation (PSE), a technique preferred over traditional logistic regression in binary response scenarios, as it addresses the bias created by confounding variables in nonrandomized studies [21]. PSE techniques include propensity score matching (PSM), inverse probability weighting, stratification, and covariance adjustment. Stratification and regression adjustment yield conditional effect estimates, which precede obtaining potential outcomes for marginal effect estimates. In contrast, matching and inverse probability weighting allow for the direct determination of marginal effect estimates [22,23]. The average treatment effect for the treated (ATT), a type of marginal estimate, can be directly derived through matching [24]. The use of propensity scores [25,26] to balance treated and control groups on observed characteristics is increasingly common in medical, epidemiological, and social science research. It approximates an experimental design where random assignment ideally balances all confounding variables. Some applications include assessing the effect of teenage alcohol use on educational attainment [27], and investigating the effects of small school size on mathematics achievement [28], Tamma et al. [29] also employed matching to compare clinical outcomes of oral step-down versus continued intravenous therapy in patients with Enterobacteriaceae Bacteremia. Another cancer research study found that propensity score matching efficiently creates matched case-control groups from large cohort studies, enhancing confidence in statistical inferences [30]. In this study, we evaluate the effect of veteran status (treatment) on SCD (outcome) using the 1:1 nearest neighbor propensity score matching method, suitable for datasets with significantly larger control groups than treatment groups [19]. Although PSM assists in reducing bias by balancing observed covariates between treatment and control groups in quasi-experimental design, it is still subject to potential residual confounding from unmeasured, imperfectly measured, or omitted covariates in the matching process or in model adjustment [31–33]. This limitation is especially relevant for the BRFSS data used in our SCD study, since this survey did not measure some factors possibly associated with SCD such as physical and moral injuries, combat engagement status, PTSD, TBI, environmental exposures, and lifestyles after discharge. However, a good balanced matching can strengthen inferences about the association between veteran status and SCD status, where a balanced matching is evaluated using the propensity distribution and the standardized mean difference [22,34]. Our PSM analysis lays a foundation for future rigorous causal analysis of the effect of military-related activities on subjective cognitive decline.

The remaining sections are comprised as follows. Section 2 describes the study population, variables, data pre-processing, and statistical methodology for PSM. Section 3 reports the characteristics of the study data, describes variable selection, propensity score matching procedure, and the results of the logistic regression analyses before and after PSM. Section 4 discusses the main findings and limitations of this study and concludes with potential future research directions.

## Methodology

### Study population

We obtained our analytical data from the 2019 Behavioral Risk Factor Surveillance System (BRFSS) Survey Data [35] and Documentation [36], which is a widely used data source for promoting health and health policy, collects behavioral health risk data annually using the noninstitutionalized 18 years or older adult population at the state and local levels across the United States and territories. BRFSS is a deidentified, anonymized data set for public use, and the Institutional Review Board waived consent and approved our retrospective study of the CDC public data with exempt status. For our study, we utilized the SCD module data, which surveys participants aged 45 years or older whether they have experienced confusion or memory loss that has become more frequent or worsened in the past 12 months at the surveying time. Because of data availability, we considered White, Black or African-American, and Hispanic or Latin-American male population among the SCD module participants. In order to explore the impact of veteran status on SCD among these participants, we considered a total of 32 observed variables, as indicated by previous studies [37]. These variables include six socio-demographic factors, seven health-related variables, nine behavioral factors, and ten comorbidities. The respondents were distributed across 49 states in the United States, the District of Columbia, and two U.S. territories (Guam and Puerto Rico). The data collection period spanned over 12 months in 2019.

### Variable definition

#### Subjective Cognitive Decline (SCD)

Participants were classified as having subjective cognitive decline if they answered ‘yes’ to the question, “During the past 12 months, have you experienced confusion or memory loss that is happening more often or is getting worse?”A ‘no’ response indicated the absence of SCD. This dichotomous measure, reflecting the SCD status of participants, was used as the response or outcome variable in our analysis.

#### Veteran status

Participants who responded ‘yes’ to the question “Have you ever served on active duty in the United States Armed Forces, either in the regular military or in a National Guard or military reserve unit?” were classified as veterans. This variable was considered to represent the treatment effect in our analysis.

#### Observed variables

Based on prior research [1,37], six socio-demographic variables were selected: race/ethnicity, age group, income level, education level, employment status, and marital status. Additionally, a review of the literature led to the inclusion of nine behavioral factors: smoking status, heavy drinking status, exercise level, fruit consumption level, vegetable consumption level, oral tobacco consumption, difficulty walking or climbing stairs, difficulty dressing or bathing, and difficulty doing errands alone; seven health-related factors: general health, physical health, mental health, health coverage, body mass index, routine medical check-up, and unaffordability of medical cost; and ten comorbidities: hypertension, cholesterol level, coronary heart disease, stroke, kidney disease, diabetes, asthma, cancer, blindness, and arthritis [1,4,5,9]. All 32 variables were assessed using a standardized questionnaire administered through landline and cell phone interviews. Refer to the 2019 BRFSS Document for the detailed description of the treatment and response variables, response variables, and 32 covariates [35].

#### Propensity score matching

The estimated propensity score, as defined by Rosenbaum and Rubin (1983) [25], is the predicted conditional probability of assignment to a specified treatment, *z*_*i*_, given a vector of observed covariates (***x***_*i*_). For subject *i*, *i* = 1,2,⋯,*N*, the propensity score, denoted by *e*(***x***_*i*_), is given as:

$$e(x_{i})=Pr({z_{i}=1|x}_{i}),$$ (1)

where 0<*e*(***x***_*i*_)<1, *z*_*i*_ = 1 is for treatment, *z*_*i*_ = 0 is for control, and *x*_*i*_ is a set of measured covariates given as ***x***_*i*_ = (*x*_1_, *x*_2_,…,*x*_*p*_). To estimate the propensity score, a logistic regression model is used, where the binary treatment effect (coded as yes = 1 and no = 0) is regressed on the subjects’ baseline characteristics (covariates). Logistic regression is particularly effective for summarizing the influence of confounders on the probability of exposure in cases where the outcome is rare [38]. In a logistic regression, Eq (1) can be written as

$$e(x_{i})=\frac{e^{\beta_{0}+\beta_{1}x_{1}+\cdots +\beta_{p}x_{p}}}{1+e^{\beta_{0}+\beta_{1}x_{1}+\cdots +\beta_{p}x_{p}}},$$

and the logit function is derived as

$$\log\left(\frac{e(x_{i})}{1-e(x_{i})}\right)=\beta_{0}+\beta_{1}x_{1}+\cdots +\beta_{p}x_{p},$$

where the coefficients, *β*_0_, *β*_1_,⋯,*β*_*p*_ are estimated via a maximum likelihood or generalized linear model.

Propensity score estimation addresses the issue of treatment selection bias in observational studies. This method enables nonrandomized studies to simulate certain characteristics of a randomized controlled trial (RCT) [19,31]. The process involves two steps: (1) Designing the study as a simulated randomized experiment, based solely on participant characteristics without considering the outcome, and (2) Analyzing the outcomes to compare differences between treated and control groups. Key considerations in estimating propensity scores include model selection and specification, fitting, balance of covariates, and confirmation of overlap. This approach aims to estimate the treatment effect on outcomes while controlling for confounding variables [21]. In cases of sparse data, good overlap between treated (exposed) and untreated (unexposed) groups aids in identifying model misspecifications. Achieving reduced residual variance depends on including the correct covariates, particularly those that affect either the treatment, the outcome, or both, and incorporating necessary interaction terms among these covariates. Controlling multiple confounders is crucial in the propensity score process [38]. Although propensity score matching has limitations such as biased estimation caused by imbalanced matching [39], there are desirable properties such as equal percent bias reduction (EPBR) [25,40,41]. As Wang [42] pointed out, we incorporate the PSM method because we have 32 categorical covariates under the condition of a proper matching, which will be analyzed in Section 3.4.

PSM is designed to align the distribution of observed baseline covariates between treated and control groups, enabling an efficient comparison of potential outcomes. Various matching techniques include optimal pair matching, nearest neighbor matching, optimal full matching, genetic matching, coarsened exact matching, and subclassification. Among these, one-to-one nearest neighbor matching is frequently utilized in PSM [19,24]. We examined the impact of current or past military service on individuals with subjective cognitive decline, matching was employed to create comparable groups between treated (veterans) and control (non-veterans) subjects. This method is particularly appropriate when the exposed group is smaller than the control group, as indicated in Section 2.4. Should the situation be reversed, the inverse probability of treatment weighting would be more suitable.

In order to evaluate the balance in the matched sample, standard statistical significance testing is inappropriate as it relates to a hypothetical superpopulation and is influenced by sample size [25]. Instead, balance assessment should be intrinsic to the matched sample and not affected by its size [22]. Standardized mean differences (SMD) are commonly recommended for this purpose, with a difference under 10% considered indicative of reasonable balance between treatment groups [22,23,34,43]. This metric uses the pooled standard deviation to compare mean differences between exposed and unexposed groups. For a dichotomous variable, the SMD denoted by *d*, is defined below,:

$$d=\frac{\left({\hat{p}}_{treatment}-{\hat{p}}_{control}\right)}{\sqrt{\frac{{\hat{p}}_{treatment}(1-{\hat{p}}_{treatment})+{\hat{p}}_{control}(1-{\hat{p}}_{control})}{2}}},$$ (2)

where ${\hat{p}}_{treatment}$ and ${\hat{p}}_{control}$ are the mean or prevalence of the binary variable among the treated and control groups, respectively [19].

Visual tools such as density plots or histograms are also useful for illustrating the distribution and balance between the full sample and matched sample. While the PSM may not be able to compensate for poor data quality or limited study design, it is valuable in situations where RCTs are not feasible. PSM prevents overfitting and inflated type-1 error rates by ensuring covariate balance and overlap, independent of the outcome variable [20].

#### Data description and pre-processing

We extracted a total of 32 observed covariates, along with a response variable (SCD) and a treatment variable (veteran status) from the 2019 BRFSS data. This resulted in a dataset comprising 418,268 observations and 34 variables. Within this dataset, 12,367 participants responded “yes” to having SCD, 98,808 participants responded “no,” and there were 307,093 instances of “missing/no response” in the SCD module. The initial step in our data processing involved checking for missing values across all variables. We found that 22 variables had approximately 0% missing values, 11 variables had less than 9% missing values, and the response variable (SCD) had the highest percentage of missing values at 73%. In order to refine the dataset further, we applied specific inclusion-exclusion criteria, such as retaining only responses from male participants and those identifying as Black or African-American, White, or Hispanic or Latin-American. Advanced filtering techniques were used to eliminate all missing values, including responses categorized as “Refused” and “Not asked/Missing.” These criteria were informed by the consideration that small sample sizes in certain categorical levels could lead to low statistical power, which is generally undesirable in statistical analysis.

Notably, the SCD module in the BRFSS 2019 survey was only administered to participants aged 45 years or older. After we applied these rules and filters, our dataset was reduced to 32,431 observations, which comprised of 3,373 (10.4%) participants who responded “yes” for SCD and 29,058 (89.6%) participants who responded “no.” Regarding the treatment variable, the dataset included 10,685 (32.9%) veterans and 21,746 (67.1%) non-veterans. Next, we processed the re-categorization of certain categorical variables, as outlined in the 2019 BRFSS LLCP Codebook Report [35] and its supplementary materials. For example, the income level variable, originally featuring eight subcategories, was reclassified into four distinct groups for clarity and ease of analysis. This re-categorization process was applied systematically to each specified variable, followed by the reordering of these variables based on predefined reference levels. These reference levels were selected based on our research objectives and established normative categories in the field.

**Fig 1:**
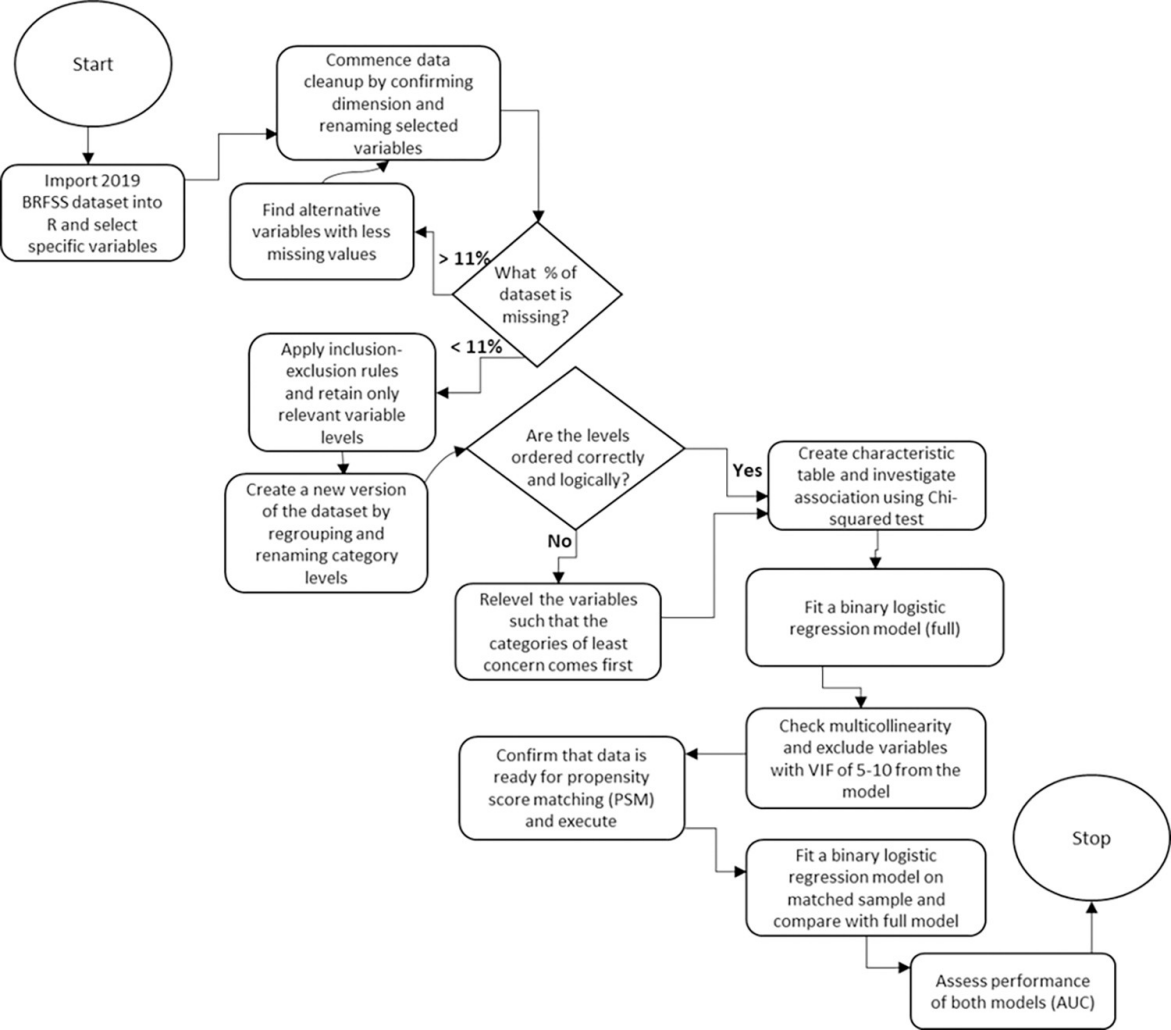
A flow chart of data preprocessing and analysis using the 2019 Behavioral Risk Factor Surveillance System (BRFSS) dataset including the 2019 cognitive decline module.

Subsequently, we constructed a baseline characteristic table using counts and frequencies to describe the distribution of each variable in relation to the outcome variable, SCD. Last, we applied PSM-related logistic regression to model our dataset, as detailed in our methodology. Fig 1 provides a comprehensive overview of the sequential steps undertaken in the preparation, processing, and analysis of our dataset.

## Results

### Characteristics table of study sample

In Table 1A and 1B, we detailed the frequency and percentage of individuals with or without SCD across the socio-demographic and behavioral, health-related, and comorbid factor variables. The prevalence rate of SCD in our study population is approximately 10.4%, with 3,373 cases of SCD out of 32,431 observations. The veteran status, our primary treatment variable, is linked with a higher prevalence rate of SCD, with 12.6% among veterans compared to 9.3% among non-veterans. Regarding race/ethnicity, the Hispanic or Latin-American group has the highest prevalence rate of SCD at 12.6%, compared to the Black or African-American (10.6%) and White (10.3%) groups. Age also plays a significant role, as participants aged 65 or older exhibit a 2.3% higher prevalence rate of SCD compared to their younger counterparts.

**Table 1:** a: Socio-demographic and behavioral characteristics of male adults aged 45 and older as a function of subjective cognitive decline status (SCD); Behavioral Risk Factor Surveillance System Data 2019. **b:** Health-related and comorbid characteristics of male adults aged 45 and older as a function of subjective cognitive decline status (SCD); Behavioral Risk Factor Surveillance System Data 2019.

			SCD	No SCD
Type	Variable	Value	n = 3373 (10.4%)	n = 29058 (89.6%)
Socio- demographic	Veteran	No	2026 (9.3%)	19720 (90.7%)
		Yes	1347 (12.6%)	9338 (87.4%)
	Race	White	2929 (10.3%)	25566 (89.7%)
		Black	269 (10.6%)	2276 (89.4%)
		Hispanic	175 (12.6%)	1216 (87.4%)
	Age group	45 to 54	566 (8.4%)	6192 (91.6%)
		55 to 64	946 (9.5%)	8961 (90.5%)
		65 or older	1861 (11.8%)	13905 (88.2%)
	Income level	< $25K	1149 (19.1%)	4854 (80.9%)
		$25K - $50K	917 (12.5%)	6390 (87.5%)
		$50K - $75K	474 (8.6%)	5044 (91.4%)
		> $75K	833 (6.1%)	12770 (93.9%)
	Education level	No HS	356 (18.4%)	1581 (81.6%)
		Graduated HS	995 (11.9%)	7356 (88.1%)
		Attended college	933 (11.0%)	7528 (89.0%)
		Graduated college	1089 (8.0%)	12593 (92.0%)
	Employment status	Employed	913 (5.9%)	14658 (94.1%)
		Unemployed	149 (17.8%)	686 (82.2%)
		Out of labor market	681 (27.6%)	1783 (72.4%)
		Retired	1630 (12.0%)	11931 (88.0%)
	Marital status	Married	1799 (8.6%)	19190 (91.4%)
		Never married	335 (12.1%)	2423 (87.9%)
		Previously married	794 (13.5%)	5066 (86.5%)
		Widowed	445 (15.8%)	2379 (84.2%)
Behavioral	Smoking status	Never	1218 (7.8%)	14497 (92.2%)
		Former	1503 (12.0%)	10972 (88.0%)
		Current	652 (15.4%)	3589 (84.6%)
	Heavy drinker	No	3140 (10.3%)	27333 (89.7%)
		Yes	233 (11.9%)	1725 (88.1%)
	Exercise	No	1201 (14.6%)	7001 (85.4%)
		Yes	2172 (9.0%)	22057 (91.0%)
	Fruit consumption	< Once daily	1478 (11.5%)	11393 (88.5%)
		≥ Once daily	1895 (9.7%)	17665 (90.3%)
	Veg consumption	< Once daily	803 (12.5%)	5632 (87.5%)
		≥ Once daily	2570 (9.9%)	23426 (90.1%)
	Oral tobacco consumption	No	3154 (10.3%)	27447 (89.7%)
		Yes	219 (12.0%)	1611 (88.0%)
	Difficulty running errands	No	2664 (8.7%)	27902 (91.3%)
		Yes	709 (38.0%)	1156 (62.0%)
	Difficulty walking or climbing stairs	No	1895 (7.1%)	24732 (92.9%)
		Yes	1478 (25.5%)	4326 (74.5%)
	Difficulty dressing or bathing	No	2788 (9.0%)	28057 (91.0%)
		Yes	585 (36.9%)	1001 (63.1%)
** **	** **	** **	**SCD**	**No SCD**
**Type**	**Variables**	**Category levels**	**n = 3373 (10.4%)**	**n = 29058 (89.6%)**
Health related	Heath status	Excellent	161 (3.6%)	4324 (96.4%)
		Very good	616 (5.7%)	10125 (94.3%)
		Good	1056 (10.0%)	9497 (90.0%)
		Fair	904 (19.0%)	3854 (81.0%)
		Poor	636 (33.6%)	1258 (66.4%)
	Physical Health	Good (0 days)	1293 (6.2%)	19710 (93.8%)
		Bad (1–13 days)	891 (13.1%)	5931 (86.9%)
		Bad (14–30 days)	1189 (25.8%)	3417 (74.2%)
	Mental Health	Good (0 days)	1629 (6.5%)	23384 (93.5%)
		Bad (1–13 days)	838 (17.0%)	4077 (83.0%)
		Bad (14–30 days)	906 (36.2%)	1597 (63.8%)
	Body mass index	Underweight	38 (17.1%)	184 (82.9%)
		Normal weight	720 (11.0%)	5853 (89.0%)
		Overweight	1295 (9.3%)	12597 (90.7%)
		Obese	1320 (11.2%)	10424 (88.8%)
	Health coverage	No	202 (12.3%)	1442 (87.7%)
		Yes	3171 (10.3%)	27616 (89.7%)
	Routine medical check-up	≤ 2 years	3205 (10.6%)	26962 (89.4%)
		> 2 years	168 (7.4%)	2096 (92.6%)
	Unafford Medical Cost	No	2873 (9.5%)	27397 (90.5%)
		Yes	500 (23.1%)	1661 (76.9%)
Cormobid	Hypertension	No	1193 (8.1%)	13582 (91.9%)
		Yes	2180 (12.3%)	15476 (87.7%)
	High cholesterol	No	1367 (8.1%)	15569 (91.9%)
		Yes	2006 (12.9%)	13489 (87.1%)
	Coronary heart disease	No	2460 (9.0%)	24901 (91.0%)
		Yes	913 (18.0%)	4157 (82.0%)
	Stroke	No	2918 (9.6%)	27538 (90.4%)
		Yes	455 (23.0%)	1520 (77.0%)
	Kidney disease	No	3061 (9.9%)	27719 (90.1%)
		Yes	312 (18.9%)	1339 (81.1%)
	Diabetes	No	2290 (9.2%)	22712 (90.8%)
		Yes	1083 (14.6%)	6346 (85.4%)
	Asthma	Never	2888 (9.8%)	26568 (90.2%)
		Former	146 (13.6%)	929 (86.4%)
		Current	339 (17.8%)	1561 (82.2%)
	Cancer	No	2809 (9.9%)	25590 (90.1%)
		Yes	564 (14.0%)	3468 (86.0%)
	Blindness	No	2896 (9.4%)	27842 (90.6%)
		Yes	477 (28.2%)	1216 (71.8%)
	Arthritis	No	1456 (7.2%)	18677 (92.8%)
		Yes	1917 (15.6%)	10381 (84.4%)

*Subjective Cognitive Decline (SCD) status: Self-reported experience with confusion or memory loss that is happening more often or getting worse in the last 12 months.

Furthermore, the prevalence rate of SCD is significantly higher in individuals facing specific challenges or health conditions. For example, those unable to afford medical costs have a prevalence rate of 23.1%, and individuals with difficulty dressing have a remarkably high rate of 36.9%. Similarly, the prevalence is 25.5% for those with difficulty walking and 38.0% for individuals with difficulty in running errands. The results also show higher prevalence rates of SCD among stroke patients (23.0%), blind adults (28.2%), and adult participants who experienced poor mental or physical health for 14–30 days (36.2% for bad mental health and 25.8% for bad physical health). These findings suggest a strong association between these aforementioned conditions and a higher prevalence of SCD.

#### Variable selection

We applied the Bayesian Information Criterion (BIC) to perform stepwise variable selection on the variables by specifying the treatment variable (veteran status) and the six socio-demographic factors (age group, race, employment status, income level, marital status, education level) as the minimum set of predictors to be retained by the stepwise procedure. The actual selection was performed on the full model, which includes the remaining 26 variables, comprising behavioral factors, comorbidities, and health-related factors. Our decision to retained socio-demographic variables as baseline covariates and apply BIC to other factors was informed by a thorough review of relevant literature [1,15–18]. Our model selected a total of 11 variables for Black or African-American males, whereas 18 variables were selected for the all male population.

For Black or African-American males, the selected variables included one health-related factor (mental health status), two behavioral factors (difficulty walking and difficulty running errands), and two comorbidities (arthritis and blindness). These selected behavioral factors appear to have a close link with the chosen health-related variable, which is mental health status. This connection, though indicative, would require further clinical diagnosis for confirmation. In contrast, for the all male population (White, Black or African-American, and Hispanic or Latin-American males) in this study, the BIC identified additional health-related factors, including physical health status, general health status, and the unaffordability of medical costs. The supplementary behavioral variable for this group was difficulty dressing. In addition, the comorbidities incorporated into the model were high cholesterol, and stroke. This broader range of selected variables for the all male population suggests a more complex interplay of factors influencing the outcome of interest compared to the subset of Black or African-American males. For example, variables such as the inability to afford medical costs, absent in the Black or African-American male group, were pertinent for the all male population. Using these selected variables, we fitted a full binary multivariable logistic model. After verifying the absence of multicollinearity and examining interactions between specific variables (veteran status, age, and race), we proceeded with propensity score estimation.

#### Propensity Score Matching (PSM) analysis

**Fig 2:**
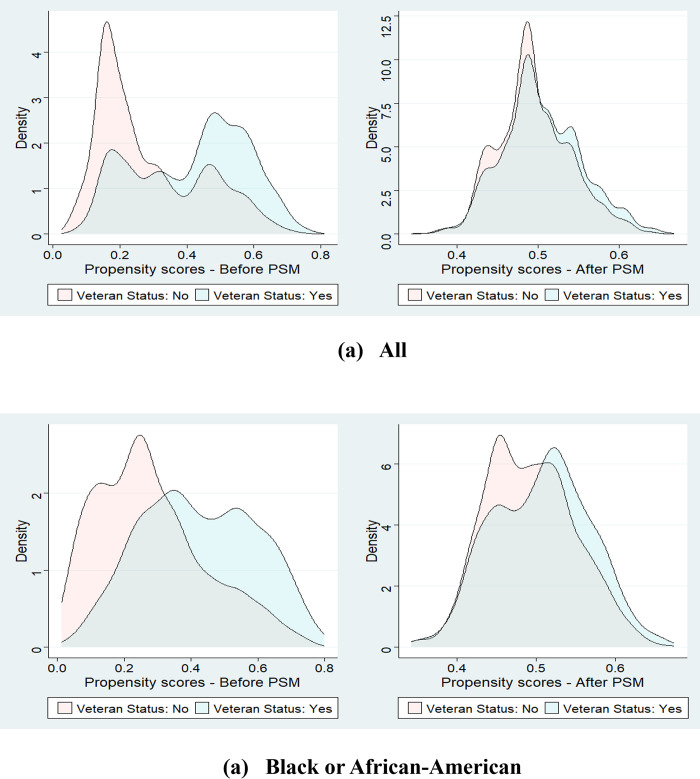
(a) The propensity score distribution for all racial/ethnicity groups and (b) The propensity score distribution for the Black or African-American groups. The left density plot shows before the PSM with the full sample, and the right density plot shows after PSM with the matched sample of veterans and non-veterans. The blue-color and peach-color density plots represent veterans and non-veterans, respectively.

We calculated propensity scores for the binary veteran status using the aforementioned selected socio-demographic, behavioral, health-related, and comorbid factors in the logistic regression. These scores, as defined in Eq (1), represent each participant’s predicted probability of being a veteran, with values ranging from 0 to 1. Fig 2A shows the distribution of propensity scores for male veterans (treated group) and non-veterans (control group) in both the full and matched samples, while Fig 2B displays a similar distribution for Black or African-American male veterans and non-veterans. Before implementing PSM, most participants identified as veterans had likely higher propensity scores, ranging from 0.45 to 0.65, indicating a greater probability of being veterans. In contrast, most subjects in the non-veteran group had lower propensity scores, predominantly less than 0.4. This imbalance was rectified in the matched sample groups, where the distribution of propensity scores became more balanced between the veteran and non-veteran groups. In Fig 2A and 2B, the right side plots display considerable overlap in the propensity score distributions of the treated and control groups, signifying the effectiveness of our approach in equalizing these distributions.

**Fig 3:**
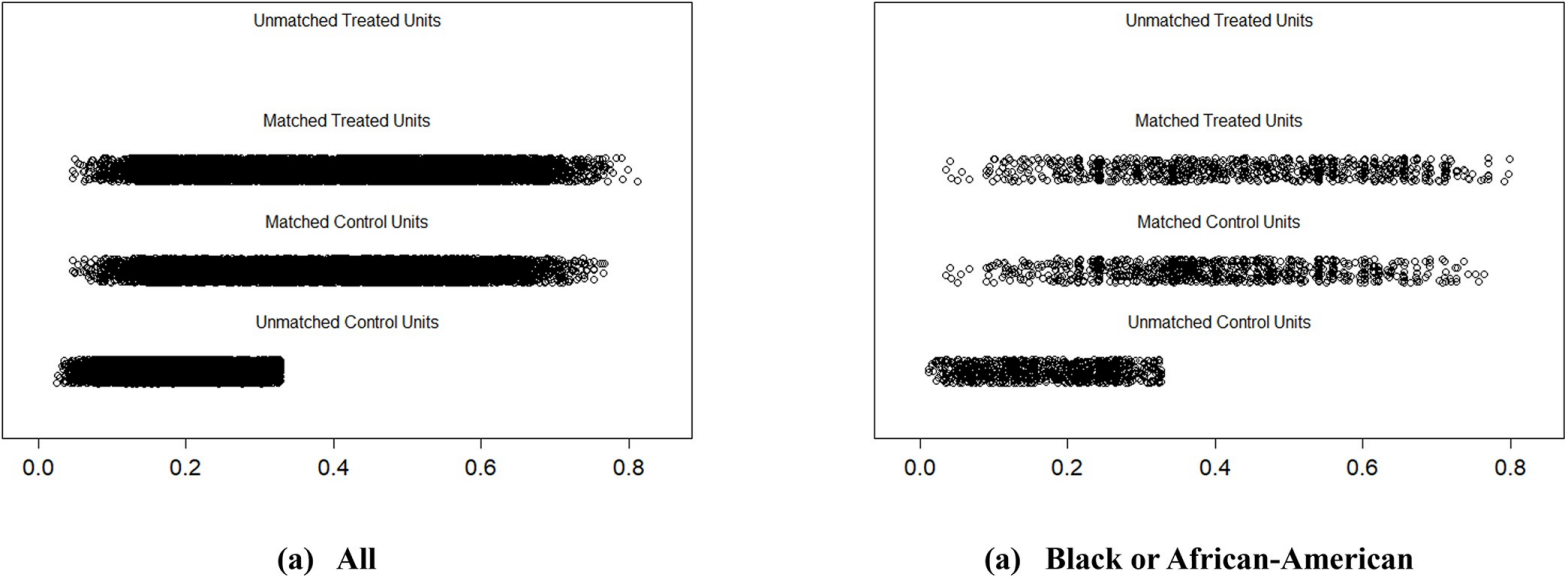
Distribution of propensity scores between matched and unmatched treated (veteran) and control (non-veteran) units. (a) All (White, Black or African-American, and Hispanic or Latin-American) males and (b) Black or African-American males. No unmatched treated units are displayed due to unbalanced data.

We employed the method of propensity score estimation for matching non-veterans to veterans based on their propensity score values. In Table 2, the standardized mean differences resulting from the 1:1 nearest neighbor propensity score matching without replacement are presented. Table 2 shows that the 1:1 nearest neighbor propensity score matching achieved a reasonable balance across each covariate, effectively reducing treatment selection bias. This 1:1 matching method achieved a better balance compared to other techniques we used, such as optimal pair and exact matching. Fig 3A further demonstrates this matching process, where similar male non-veterans were paired with corresponding veterans, resulting in a matched sample consisting of 10,685 veterans and an equal number of non-veterans, with 11,061 non-veterans remaining unmatched. Similarly, Fig 3B depicts the pairing of 834 Black or African-American male non-veterans with an equal number of Black or African-American male veterans, leaving a total of 877 Black or African-American male non-veterans in the unmatched group. This matching approach ensures a more balanced comparison between the veteran and non-veteran groups in our study.

**Table 2:** Standardized mean differences between the treated (veterans) and control (non-veterans) groups for before and after propensity score matching using the 1:1 nearest neighbor method of matching.

		Before PSM		After PSM	
Variables	Category levels	All	Black or African-American	All	Black or African-American
Race	White	0.07		0.00	
	Black	0.00		0.00	
	Hispanic	-0.12		0.00	
Age group	45 to 54	-0.41	-0.26	-0.03	-0.04
	55 to 64	-0.49	-0.15	-0.03	-0.05
	65 or older	0.70	0.34	0.05	0.08
Income level	< $25K	-0.06	-0.38	-0.01	-0.05
	$25K - $50K	0.15	0.11	0.04	0.03
	$50K - $75K	0.07	0.15	0.01	0.06
	> $75K	-0.15	0.14	-0.04	-0.03
Employment status	Employed	-0.55	-0.27	-0.10	-0.15
	Unemployed	-0.07	-0.12	0.00	0.06
	Out of labor market	-0.12	-0.19	-0.02	0.02
	Retired	0.60	0.43	0.10	0.11
Marital status	Married	-0.04	0.16	-0.05	-0.03
	Never married	-0.23	-0.42	-0.03	0.02
	Previously married	-0.02	0.01	-0.01	-0.03
	Widowed	0.23	0.12	0.11	0.06
Education level	No high school	-0.22	-0.63	-0.03	-0.01
	Graduated high school	0.01	-0.14	0.01	-0.06
	Attended college	0.16	0.31	0.07	0.05
	Graduated college	-0.08	0.11	-0.07	0.01
Difficulty running errands	No	-0.03	0.04	-0.02	-0.03
	Yes	0.03	-0.04	0.02	0.03
Difficulty walking	No	-0.15	-0.03	-0.07	-0.06
	Yes	0.15	0.03	0.07	0.06
Difficulty dressing	No	-0.02		-0.02	
	Yes	0.02		0.02	
General health status	Excellent	-0.06		-0.02	
	Very good	-0.07		-0.03	
	Good	0.06		0.02	
	Fair	0.04		0.02	
	Poor	0.02		0.02	
Physical Health	Good	-0.07		-0.03	
	Bad (1–13 days)	0.02		0.01	
	Bad (14–30 days)	0.06		0.03	
Mental Health	Good (0 days)	0.08	-0.03	-0.01	-0.05
	Bad (1–13 days)	-0.10	-0.02	0.01	0.02
	Bad (14–30 days)	0.01	0.06	0.01	0.05
Unaffordability medical cost	No	0.15		0.01	
	Yes	-0.15		-0.01	
High cholesterol	No	-0.12		-0.03	
	Yes	0.12		0.03	
Stroke	No	-0.10		-0.04	
	Yes	0.10		0.04	
Blindness	No	-0.04	0.13	-0.02	0.00
	Yes	0.04	-0.13	0.02	0.00
Arthritis	No	-0.18	-0.17	-0.04	-0.08
	Yes	0.18	0.17	0.04	0.08

* The standardized mean difference is not available for Race in the Black or African-American only. The standardized mean differences for covariates are not available in the Black or African-American only due to the variable selection.

After the matching process, we utilized Eq (2) to calculate the standardized mean differences for each covariate between veterans and non-veterans. According to Table 2, post-matching covariates displayed a standardized mean difference of less than 10% between the veteran and non-veteran groups. The only exceptions were the widowed marital status group and the retired group in the employment category, which showed marginal differences. Before matching, however, several variables—including age group, employment status, education level, marital status, unaffordability of medical cost, arthritis, difficulty walking or climbing stairs, and high cholesterol—had standardized mean differences exceeding 10%.

#### Logistic regression (before and after PSM)

In the final step of our analysis, we modeled the SCD status using the veteran status as a treatment variable and the selected covariates for the all population and Black or African-American population separately. We fitted a logistic regression model on the matched sample to estimate the treatment effect of veteran status on SCD. This analysis was previously conducted on the full, unmatched samples before the propensity score matching. Table 3 details the results of binary logistic regressions analyzing the effect of veteran status on SCD among the all male population using data before and after PSM. The table demonstrates the odds ratio (OR) and its 95% confidence interval (CI) for the treatment variable of the veteran status and other covariates. The single, two, and thee asterisks indicate a significance of the corresponding variable at 5%, 1%, and 0.1% significance level, respectively. These results show a statistically significant association between veteran status and SCD in both pre- and post-PSM analyses. The odds ratio for veteran status indicates an increased risk of SCD, changing from 1.20 before PSM to 1.16 after PSM, which means that male veterans are at least 16% more likely to experience SCD compared to non-veterans. The slightly higher odds of 1.20 in the traditional regression model (before PSM) suggests that some of the observed association could be attributable to differences in covariates between veterans and non-veterans. PSM attempts to control for these differences, potentially offering a more accurate estimate of the isolated effect of veteran status on SCD.

**Table 3:** Odds ratio (OR) of the binary logistic regression model fitted before and after propensity score matching for the all (White, Black or African-American, and Hispanic or Latin-American) participants who responded yes to the subjective cognitive decline module in BRFSS 2019 data.

		Before PSM	After PSM
Variables	Category levels	OR (95% C.I)	OR (95% C.I)
**Veteran status**	Yes	1.20 (1.10–1.31) ***	1.16 (1.06–1.27) ***
** **	*(ref*: *No)*		
**Race**	Black	0.67 (0.58–0.78) ***	0.71 (0.59–0.84) ***
** **	Hispanic	1.07 (0.89–1.29)	1.16 (0.90–1.49)
** **	*(ref*: *White)*		
**Age group**	55 to 64	1.02 (0.90–1.15)	1.02 (0.85–1.22)
** **	65 or older	1.30 (1.14–1.50) ***	1.26 (1.06–1.51) *
** **	*(ref*: *45 to 54)*		
**Income level**	< $25K	1.23 (1.07–1.41) **	1.22 (1.04–1.42) *
** **	$25K - $50K	1.24 (1.10–1.39) ***	1.26 (1.10–1.44) ***
** **	$50K - $75K	1.07 (0.94–1.22)	1.07 (0.93–1.23)
** **	*(ref*: *> $75K)*		
**Employment status**	Unemployed	1.44 (1.16–1.79) ***	1.51 (1.14–2.00) ***
** **	Out of labor market	1.39 (1.20–1.61) ***	1.22 (1.01–1.48) *
** **	Retired	1.34 (1.19–1.50) ***	1.28 (1.12–1.46) ***
** **	*(ref*: *Employed)*		
**Marital status**	Never married	0.94 (0.81–1.08)	0.84 (0.69–1.03)
** **	Previously married	0.98 (0.88–1.09)	0.91 (0.80–1.04)
** **	Widowed	1.15 (1.01–1.31) *	1.16 (1.01–1.32) *
** **	*(ref*: *Married)*		
**Education level**	No high school	1.07 (0.91–1.26)	1.04 (0.83–1.31)
** **	Graduated high school	0.95 (0.85–1.06)	0.90 (0.79–1.02)
** **	Attended college	0.98 (0.88–1.09)	0.96 (0.85–1.07)
** **	*(ref*: *Graduated college)*		
**Difficulty running errands**	Yes	1.59 (1.39–1.82) ***	1.61 (1.37–1.89) ***
	*(ref*: *No)*		
**Difficulty walking and climbing stairs**	Yes	1.43 (1.29–1.59) ***	1.48 (1.31–1.66) ***
** **	*(ref*: *No)*		
**Difficulty dressing**	Yes	1.30 (1.13–1.50) ***	1.26 (1.06–1.49) *
** **	*(ref*: *No)*		
**General health status**	Excellent	0.58 (0.49–0.69) ***	0.63 (0.51–0.77) ***
** **	Very good	0.76 (0.68–0.85) ***	0.78 (0.69–0.89) ***
** **	Fair	1.13 (1.01–1.26) *	1.13 (1.00–1.29)
** **	Poor	1.22 (1.05–1.43) *	1.21 (1.01–1.46) *
** **	*(ref*: *Good)*		
**Physical health**	Bad (1–13 days)	1.31 (1.19–1.45) ***	1.29 (1.15–1.45) ***
** **	Bad (14–30 days)	1.26 (1.11–1.43) ***	1.18 (1.02–1.37) *
** **	*(ref*: *Good)*		
**Mental health**	Bad (1–13 days)	2.45 (2.22–2.70) ***	2.45 (2.18–2.76) ***
** **	Bad (14–30 days)	4.06 (3.62–4.55) ***	3.88 (3.39–4.44) ***
** **	*(ref*: *Good (0 days))*		
**Unaffordability medical cost**	Yes	1.46 (1.29–1.66) ***	1.45 (1.22–1.72) ***
	*(ref*: *No)*		
**High cholesterol**	Yes	1.26 (1.16–1.36) ***	1.21 (1.10–1.33) ***
** **	*(ref*: *No)*		
**Stroke**	Yes	1.39 (1.23–1.58) ***	1.39 (1.21–1.60) ***
** **	*(ref*: *No)*		
**Blindness**	Yes	1.48 (1.29–1.69) ***	1.42 (1.21–1.66) ***
** **	*(ref*: *No)*		
**Arthritis**	Yes	1.32 (1.22–1.44) ***	1.32 (1.20–1.45) ***
** **	*(ref*: *No)*		

* (ref: level) denotes the reference level for each categorical covariate; PSM: Propensity score matching; A (B-C) denotes Odds ratio (95% Confidence interval); *, **, and *** denote that the P-value is significant at 5, 1%, and 0.1% significance levels, respectively.

Ethnicity-based comparisons revealed that, compared to White males, Black or African-American males experience a 29% lower risk of SCD after PSM (OR:0.71, 95% CI:0.59–0.84), which was regardless of the veteran status. Interestingly, most other factors showed similar significance before and after PSM. Specifically, the SCD status is significantly associated with behavioral factors (such as difficulty running errands and difficulty walking and climbing stairs), health related factors (general, physical, and mental health status, and unaffordability of medical costs), and comorbidities (blindness, arthritis, and stroke) both before and after PSM.

Table 4 presents the results of logistic regressions analyzing the effect of veteran status on SCD among Black or African-American males before and after PSM. Focusing on Black or African-American males, the veteran status shows increasingly significant associations with SCD before and after PSM. In particular, after PSM, this Black or African-American veteran group has 69% higher risk of SCD than the same racial non-veteran group (OR:1.69, 95% CI:1.16–2.45). Additionally, the SCD status is significantly associated with behavioral factors (difficulty running errands and difficulty walking and climbing stairs), mental health status, and comorbidities (blindness and arthritis), both before and after PSM. The risk increase for these factors is exceptionally high in Black or African-American males, ranging from 46% for arthritis to over 600% for poor mental health status.

**Table 4:** Odds ratio (OR) of the binary logistic regression model fitted before and after propensity score matching for Black or African American male participants who responded yes to the subjective cognitive decline module in BRFSS 2019 data.

		Before PSM	After PSM
Variables	Category levels	OR (95% C.I)	OR (95% C.I)
**Veteran status**	Yes	1.41 (1.02–1.96) *	1.69 (1.16–2.45) *
** **	*(ref*: *No)*		
**Age group**	55 to 64	0.93 (0.62–1.39)	0.80 (0.45–1.44)
** **	65 or older	0.91 (0.56–1.47)	1.20 (0.63–2.27)
** **	*(ref*: *45 to 54)*		
**Employment status**	Unemployed	1.28 (0.64–2.53)	1.42 (0.49–4.14)
** **	Out of labor market	1.03 (0.64–1.64)	0.98 (0.51–1.87)
** **	Retired	1.41 (0.89–2.22)	1.44 (0.82–2.55)
** **	*(ref*: *Employed)*		
**Income level**	< $25K	1.11 (0.65–1.90)	1.00 (0.52–1.92)
** **	$25K - $50K	1.02 (0.60–1.73)	0.85 (0.46–1.57)
** **	$50K - $75K	1.15 (0.65–2.05)	1.15 (0.61–2.15)
** **	*(ref*: *> $75K)*		
**Marital status**	Never married	0.75 (0.48–1.17)	0.37 (0.16–0.86) *
** **	Previously married	1.22 (0.86–1.73)	1.19 (0.78–1.83)
** **	Widowed	0.81 (0.48–1.36)	0.88 (0.48–1.60)
** **	*(ref*: *Married)*		
**Education level**	No high school	1.35 (0.80–2.28)	1.10 (0.48–2.52)
** **	Graduated high school	0.86 (0.55–1.36)	0.74 (0.42–1.28)
** **	Attended college	0.78 (0.50–1.23)	0.91 (0.55–1.51)
** **	*(ref*: *Graduated college)*		
**Difficulty running errands**	Yes	2.35 (1.62–3.43) ***	2.55 (1.56–4.17) ***
	*(ref*: *No)*		
**Difficulty walking and climbing stairs**	Yes	2.15 (1.51–3.04) ***	2.37 (1.52–3.70) ***
	*(ref*: *No)*		
**Mental health**	Bad (1–13 days)	3.65 (2.57–5.17) ***	3.73 (2.38–5.84) ***
** **	Bad (14–30 days)	6.14 (4.25–8.88) ***	6.68 (4.13–10.79) ***
** **	*(ref*: *Good (0 days)*		
**Blindness**	Yes	2.11 (1.44–3.11) ***	2.05 (1.19–3.55) *
** **	*(ref*: *No)*		
**Arthritis**	Yes	1.66 (1.21–2.27) **	1.46 (0.97–2.19)
** **	*(ref*: *No)*		

* (ref: level) denotes the reference level for each categorical covariate; PSM: Propensity score matching; A (B-C) denotes Odds ratio (95% Confidence interval); *, **, and *** denote that the P-value is significant at 5, 1%, and 0.1% significance levels, respectively.

The variables that increased the odds of SCD in male subjects included older age, retirement/unemployment, lower income level, death of spouse, blindness, arthritis, poor mental health, and difficulties in daily activities, among others. Conversely, male veterans could decrease their SCD odds by at least 24% by maintaining "very good" or "excellent" general health. Notably, compared to those with good mental health, male veterans with poor mental health for 14 to 30 days had a 388% higher risk of SCD, which rose to 668% in Black or African-American males. These findings align with prior research examining SCD in different populations [1,9].

Finally, we calculated the area under the Receiver Operating Characteristic (ROC) curve (or AUC) for logistic regression analyses. The AUCs for the all male population were 0.78 and 0.76 before and after PSM, respectively. Similarly, the AUCs for the Black or African-American male population were 0.83 and 0.84 before and after PSM, respectively. These AUC values demonstrate the potential predictability of our model in distinguishing the high-risk group from the low-risk group in SCD.

## Discussion

In this study, we performed an association analysis between male veteran status and subjective cognitive decline (SCD) using propensity score matching on the 2019 BRFSS cross-sectional data. Overall, in the all population (White, Black or African-American, and Hispanic or Latin-American), male veterans significantly suffer from SCD more compared to non-veterans in these demographic groups regardless of propensity score matching with mostly socio-demographic and health related covariates. While the subjectivity of SCD could be viewed as a limitation in this study, our research established a groundwork for further investigation into the clinical cognitive health of veterans. We achieved these results by effectively randomizing our observational study, enhancing the comparability between veteran and non-veteran groups for SCD assessment. Propensity score estimation methods enhance the generalizability and accuracy of observational studies to compare outcomes between the treated (exposed) group and the control (unexposed) group. Both before and after propensity score matching, we found significant associations between SCD and various comorbidities, behavioral, and health-related factors. On the other hand, education and income levels showed insignificant associations with SCD, especially in the Black or African-American male veteran population. The BRFSS data did not measure some factors in the veteran population, such as PTSD, TBI, sleep deprivation, exposure to harmful substances, chronic exposure to high levels of noise, extreme environmental conditions, and physical stress/fatigue. This suggests future observational studies need to consider these important confounders.

A systematic review on the linkage of cognitive resilience to psychological stress in military personnel revealed that factors such as physical fatigue and environmental stressors can have consequences for cognitive processes, including attention and working memory [44]. The physical/mental mobility and health behavioral factors present in the BRFSS data could be proxies for the unmeasured variables, however, conducting a “Veteran-Specific Survey,” where these indicators are explicitly measured, would provide more insight into the mechanism why veterans have a higher risk of SCD.

Next, our analysis looked into the association between the Black or African-American male veterans and SCD status. As shown in Table 4, the risk for SCD disproportionately increased for Black or African-American male veterans. This discrepancy could be due to a variety of reasons, one of which is the disparity in socioeconomic factors in this minority racial group. When we closely examined the three statistically significant socioeconomic factors from our model (‘Unaffordability of medical costs’, ‘Employment status’, and ‘Income level’), we found that the proportion of Black veterans (19.0%) who could not afford medical costs when they needed to see a doctor was almost double that of White veterans (8.4%), especially for those who have SCD. Similar outcomes are also observed for Black veterans who were out of the labor market and those who earned less than $25,000 annually. Additionally, after PSM, the OR of veteran status for SCD in the full model, which includes all three racial groups, is 1.15, while solely considering the Black or African-American group, the OR of veteran status increases to 1.69. This increase is because the PSM excluded the Black or African American male non-veterans aged 64 years or below more to achieve one-to-one matching with the same age-group veterans with SCD, as can be seen in S1 Table. The elimination of younger Black male veterans after PSM resulted in the higher odds ratio of 1.69 for veteran status with respect to SCD in the Black-only model, as previous studies such as Olivari et al. [10] corroborates that the older the population, the higher the risk of SCD. Black or African-American veterans disproportionately suffer more from PTSD and TBI compared to White veterans, which is highly associated with neurological disorders including Alzheimer’s disease [45,46]. Dismuke et al. [47] reported that non-Hispanic Black veterans have a 44% higher likelihood of being diagnosed with moderate or severe TBI than non-Hispanic White veterans. Another study discovered that African American veterans had significantly higher PTSD scores compared to Hispanic and Caucasian veterans with similar combat experiences, suggesting different life experiences may contribute to these differences [45]. Our finding adds another potential disparity factor potentially associated with neurological disorder for Black or African-American male veterans, which warrants further investigation incorporating relevant confounders in a randomized controlled design.

The findings of this study can assist healthcare researchers and practitioners in identifying risk factors that could be utilized to promote cognitive risk reduction behaviors. It is worth mentioning some known risk factors in addition to the main variable of interest, which is veteran status. As the education level increases, the SCD prevalence rate decreases, as shown in Table 1a, which is consistent with the findings of Wooten et al. [48]. However, the association between education level and SCD is not statistically significant in the logistic regression analysis presented in Tables 3 and 4. Although there are racial differences in SCD prevalence rates—with Asian or Pacific Islander adults having the lowest rate, American Indian or Alaska Native adults having the highest prevalence rate, and White, Black or African-American, and Hispanic or Latin-American adults displaying similar prevalence rates between 10% and 11%—our study was unable to include Asian or Pacific Islander and American Indian or Alaska Native adults due to a limited number of veterans within these categories. Interestingly, variables related to physical mobility are significantly associated with SCD status in both the entire study population and the Black or African-American population, as shown in Tables 3 and 4. These variables include difficulty running errands, difficulty walking and climbing stairs, blindness, and arthritis. The discovery of these associations highlights the need for further investigation to uncover their causal relationships and to prevent the progression of SCD or other diseases.

Although our analysis between the veteran status and the SCD status demonstrated significant results, there are some limitations which could be addressed in future studies. Propensity score matching was used in this study because we considered relatively many covariates with a large sample size, and the matching between the two treatment groups was successful. However, the PSM method did not use a considerable portion of samples in the unbalanced treatment which led to many covariates’ insignificance in variable selection. Therefore, future studies can consider different causal effect estimation methods for observational studies such as doubly robust estimator (DRE). DRE does not lose unmatched samples and is a consistent estimator as long as one of the outcomes and the treatment models is correctly specified. Second, the choice of variables in a propensity score model influences the bias, variance, and mean-squared error of the estimated treatment effect [21]. Backward and forward stepwise variable selection methods, such as AIC and BIC, are commonly used in traditional low-dimensional data [49]. However, in high-dimensional data with potential for numerous confounders, more sophisticated methods are preferred over traditional methods. For example, techniques such as penalized modified objective function estimators [50], Bayesian adjustment for confounding [51], and model averaged double robust estimators [52] have been proposed for more effectively addressing confounding in covariate selection. Koch et al. [53] introduced the group lasso and doubly robust estimation (GLiDeR) approach, combining adaptive group LASSO with doubly robust estimation, for simultaneous confounder identification and causal effect estimation. Finally, although our study bellwethers the risk of male veterans’ subjective cognitive decline, we need further efforts to uncover more clinical, etiological, and epidemiological mechanisms from military service to cognitive decline. Future studies can focus on a long-term temporal causal relationship between military service and cognitive decline, in particular, for the Black or African-American veteran population.

## Supporting information

S1 Table Distribution of age groups for veteran status, race, SCD status before and after 1:1 propensity matching for veteran status. (PDF)
