# Supplementary material for: Evaluating disparity of subjective cognitive decline between male veterans and non-veterans in the United States using propensity score matching estimation: A behavioral risk factor surveillance system survey cross-sectional study
Source: PLoS One. 2024 Sep 13;19(9):e0310102. doi: 10.1371/journal.pone.0310102 (PMC11398683; doi:10.1371/journal.pone.0310102)
Supplement: S1 Table — (PDF) [file pone.0310102.s001.pdf]

Supporting Information

S1 Table. Distribution of age groups for veteran status, race, SCD status before and after 1:1 propensity matching for veteran status.

Before PSM	Veteran						Non-Veteran					
	White		Black		Hispanic		White		Black		Hispanic	
Age Group	No SCD	SCD	No SCD	SCD	No SCD	SCD	No SCD	SCD	No SCD	SCD	No SCD	SCD
45-54	953 (11.4%)	100 (8.4%)	134 (18.4%)	20 (19.0%)	60 (22.9%)	13 (26.5%)	4177 (24.3%)	352 (20.3%)	449 (29.0%)	39 (23.8%)	419 (43.9%)	42 (33.3%)
55-64	1394 (16.7%)	195 (16.3%)	225 (30.9%)	27 (25.7%)	64 (24.4%)	12 (24.5%)	6408 (37.2%)	595 (34.3%)	566 (36.6%)	69 (42.1%)	304 (31.9%)	48 (38.1%)
≥65	6000 (71.9%)	898 (75.3%)	370 (50.8%)	58 (55.2%)	138 (52.7%)	24 (49.0%)	6634 (38.5%)	789 (45.4%)	532 (34.4%)	56 (34.1%)	231 (24.2%)	36 (28.6%)
After PSM	Veteran						Non-Veteran					
	White		Black		Hispanic		White		Black		Hispanic	
Age Group	No SCD	SCD	No SCD	SCD	No SCD	SCD	No SCD	SCD	No SCD	SCD	No SCD	SCD
45-54	953 (11.4%)	100 (8.4%)	134 (18.4%)	20 (19.0%)	60 (22.9%)	13 (26.5%)	1006 (11.8%)	96 (9.6%)	127 (17.2%)	15 (17.2%)	68 (25.7%)	8 (19.0%)
55-64	1394 (16.7%)	195 (16.3%)	225 (30.9%)	27 (25.7%)	64 (24.4%)	12 (24.5%)	1595 (18.7%)	175 (17.5%)	173 (23.4%)	23 (26.4%)	48 (18.1%)	8 (19.0%)
≥65	6000 (71.9%)	898 (75.3%)	370 (50.8%)	58 (55.2%)	138 (52.7%)	24 (49.0%)	5948 (69.6%)	731 (73.0%)	440 (59.5%)	49 (56.3%)	149(56.2%)	26 (61.9%)
